# Notes and News

**Published:** 1905-01-28

**Authors:** 


### NOTES AND NEWS.

H.R.H. the Prince of Wales, Grand President, has, during the past twelve months, sanctioned (subject to inspection) the payment of the following grants to hospitals from the League of Mercy:—Royal Berkshire Hospital, £100; Holmdale Cottage Hospital, Sevenoaks, £50; Cheshunt Cottage Hospital, £25; Watford District Hospital, £25; Faversham Cottage Hospital, £20; Princess Alice Memorial Hospital, Eastbourne, £20 (two grants); Victoria Hospital, Folkestone, £20 (two grants); General Kent and Canterbury Hospital, £10 10s.; Tunbridge Wells Homœopathic Hospital and Dispensary, £10; Brentford Dispensary, £5; Enfield Cottage Hospital, £5; Hanwell Cottage Hospital, £5; St. John's Hospital, Twickenham, £5.

THE death of Dr. Ambrose Birmingham, demonstrator of Anatomy at the Roman Catholic University School of Medicine, Dublin, took place on Monday. Dr. Birmingham, who was only forty, was regarded as one of the most able anatomists in the sister island. He was an authority on cranio-cerebral topography, with special reference to the mastoid region of the skull, and on the anatomy of the digestive system.

THE Governors of Portsmouth Hospital have adopted the recommendation of the Management Committee—"not to allocate any legacy moneys to the Building Fund" by a majority of 16 against 11. The chairman, Sir John Baker, opposed the recommendation on the ground that the hand of the dead should not be placed upon the money which could be used for the benefit of the present generation, and he was supported by Dr. Ward Cousins, and others.

IN the course of the hearing on Tuesday of an application made to destroy 60 tons of coffee by the Chief Sanitary Inspector of Bermondsey, on the ground that it was unfit for food, Dr. Brown, the Medical Officer of Health for Bermondsey, Dr. Eyre of Guy's Hospital, and Dr. Bodmer, Public Analyst, gave it as their opinion that the coffee was unsound and unwholesome. On the other hand, Dr. Friswell and Dr. Smith declared that it was perfectly harmless. The magistrate dismissed the application.

IT was decided at a meeting of the members of the medical profession, held last Friday in Manchester, to establish a medical institution and club in the centre of the city. The capital is to be raised by the formation of a limited company, and the committee, which consists of leading representatives of the profession, were armed with power to carry out the scheme.

AT an inquest last week Dr. Wynne Baxter stated that when the special returns of the coroners suggested by the Home Secretary respecting inquests arising from the burning of infants through the wearing of cheap flannelette were issued in a few months' time, he feared that they would be very alarming. He himself held 73 inquiries during 1904 into infant fatalities from fire, most of which were attributable to the inflammable nature of flannelette.

AT the meeting of the Metropolitan Water Board, last week, the question of the appointment of an examining medical officer came up for consideration. It was stated in the report of the General Purposes Committee that 106 applications had been received, and that three candidates had been selected to appear before the Board that day. But the report was withdrawn because the Chairman of the Committee informed the Board that information came to their knowledge after the report had been drawn up which they ought to have the opportunity of investigating.

LAST Tuesday, at the fifth annual meeting of the Royal London Ophthalmic Hospital Guild, those who were present had the pleasure of receiving a very satisfactory report. It showed that the membership and the work of the guild were steadily increasing. During the past 12 months the guild has helped the hospital with blankets, coverlets, and sheets for the beds, and for the past two years has maintained a cot and woman's bed in the wards, while through its assistance the nurses' library had been replenished. The formation of similar guilds in connection with other hospitals is much to be desired, as they serve to encourage and stimulate personal service among the laity, while helping to preserve that individuality which is so important to every charitable institution.

THE publication of "cancer-cures" is one of the features of the day, and by this time, no doubt, the most credulous person has learned to regard them all with the deepest suspicion. But lest good corn may be lost with the chaff, it is necessary to record every claim which appears to have a scientific foundation. This is certainly the case with an anti-carcinoma serum which is stated to confer immunity in mice. Last year Dr. Gaylord obtained from Dr. Jensen, of Copenhagen, some white mice which had been inoculated with cancer, and had growing tumours. Dr. Gaylord took these mice to America, and with Messrs. Clowes and Baeslach commenced to inoculate other mice for purposes of observation. It was found that in some of the inoculated mice the tumours, after growing to a certain size, underwent retrogression, and finally disappeared. Further, it was discovered that the serum of these mice was capable of checking virulent growths in other mice and conferring immunity on them from recurrence. The observation is suggestive, and we shall await with interest a further communication on the matter.

